# Genome-wide analysis of long non-coding RNAs (lncRNAs) in two contrasting soybean genotypes subjected to phosphate starvation

**DOI:** 10.1186/s12864-021-07750-8

**Published:** 2021-06-09

**Authors:** Jinyu Zhang, Huanqing Xu, Yuming Yang, Xiangqian Zhang, Zhongwen Huang, Dan Zhang

**Affiliations:** 1 School of Life Science and Technology, Henan Collaborative Innovation Center of Modern Biological Breeding, Henan Institute of Science and Technology, Xinxiang, 453003 China; 2 Collaborative Innovation Center of Henan Grain Crops, College of Agronomy, Henan Agricultural University, Zhengzhou, 450002 China

**Keywords:** LncRNAs, Phosphate starvation, RNA-Seq, Soybean

## Abstract

**Background:**

Phosphorus (P) is essential for plant growth and development, and low-phosphorus (LP) stress is a major factor limiting the growth and yield of soybean. Long noncoding RNAs (lncRNAs) have recently been reported to be key regulators in the responses of plants to stress conditions, but the mechanism through which LP stress mediates the biogenesis of lncRNAs in soybean remains unclear.

**Results:**

In this study, to explore the response mechanisms of lncRNAs to LP stress, we used the roots of two representative soybean genotypes that present opposite responses to P deficiency, namely, a P-sensitive genotype (Bogao) and a P-tolerant genotype (NN94156), for the construction of RNA sequencing (RNA-seq) libraries. In total, 4,166 novel lncRNAs, including 525 differentially expressed (DE) lncRNAs, were identified from the two genotypes at different P levels. GO and KEGG analyses indicated that numerous DE lncRNAs might be involved in diverse biological processes related to phosphate, such as lipid metabolic processes, catalytic activity, cell membrane formation, signal transduction, and nitrogen fixation. Moreover, lncRNA-mRNA-miRNA and lncRNA-mRNA networks were constructed, and the results identified several promising lncRNAs that might be highly valuable for further analysis of the mechanism underlying the response of soybean to LP stress.

**Conclusions:**

These results revealed that LP stress can significantly alter the genome-wide profiles of lncRNAs, particularly those of the P-sensitive genotype Bogao. Our findings increase the understanding of and provide new insights into the function of lncRNAs in the responses of soybean to P stress.

**Supplementary Information:**

The online version contains supplementary material available at 10.1186/s12864-021-07750-8.

## Background

In general, long noncoding RNAs (lncRNAs) refer to transcripts longer than 200 nucleotides and do not encode open reading frames (ORFs) [1]. In eukaryotes, most lncRNAs are transcribed by RNA polymerase II and have a structure similar to that of mRNA, which includes 5′ capping, splicing and polyadenylation [2]. A growing body of evidence shows that lncRNAs play important functional roles in diverse biological processes, such as epigenetic regulation, cell cycle regulation, cellular growth and differentiation, by regulating the level of target genes [3, 4]. LncRNAs are involved in a wide range of regulatory mechanisms that impact gene expression, including chromatin remodeling, modulation of alternative splicing, fine-tuning of miRNA activity, and the control of mRNA translation or accumulation [5].

Recent advances in biological technologies, such as tiling arrays and RNA deep sequencing (RNA-seq), have made it possible to survey the transcriptomes of many organisms to an unprecedented degree [6]. LncRNAs have been widely identified in various plants, such as *Arabidopsis thaliana* [7, 8], rice [9], *Zea mays* [10] and cotton [11]. Emerging studies have revealed that lncRNAs play important roles in various biological processes, including flowering regulation [12], photomorphogenesis [13], stress responses [14, 15] and other important developmental pathways [16, 17]. For example, the rice-specific lncRNA *LDMAR* has been identified as a key gene in controlling photoperiod-sensitive male sterility [18].

Plants possess an elaborate physiological system that responds to external abiotic stress conditions [19], including phosphorus (P) deficiency. As one of the major mineral macronutrients present in all living things, P is essential for plant growth and development due to its key role in the regulation of energy metabolism and the synthesis of nucleic acids and membranes [20, 21]. Although P is abundant in soil, its direct use by plants is often limited due to its low bioavailability. Thus, low phosphorus (LP) stress represents a major limiting factor affecting plant growth and productivity [22]. P is important for plant growth and the agricultural industry, but it has been estimated that the P rock reserves will be depleted by 2050 [23]. Therefore, we need to understand the molecular mechanism underlying the responses of crops to LP stress and improve their phosphorus use efficiency. Plants have evolved numerous adaptive developmental and metabolic responses to cope with growth under phosphate-limited conditions, and these responses include modifying the root system architecture (RSA), increasing acid phosphatase activity (APA), and the release of low-molecular-weight organic acids [20]. Many studies have shown that many P-related genes, such as *GmACP1* [22], *GmHAD1* [24], and *PHR1* [25], are involved in plant growth and development. Noncoding RNAs serve as one of the key regulators involved in the P starvation response network. Changes in miRNAs, such as *miR399* [26] and *miR827* [27], constitute an important mechanism used by plants to adapt to LP environments. LncRNAs also play key roles in regulating the mRNA and/or miRNA levels of a large number of genes associated with P starvation responses [14, 28, 29], which suggests their important functions in regulating the responses of plants to LP stress. Du et al. found that *PILNCR1* (long-noncoding RNA1) can inhibit the ZmmiR399-guided cleavage of *ZmPHO2*, and the interaction between PILNCR1 and miR399 is important for the tolerance of maize to LP conditions [28].

Soybean is not only a major crop plant constituting a major agricultural industry worldwide but also an important seed crop because it is an essential source of proteins, oils and micronutrients for human and livestock consumption [30]. Because soybean seeds contain higher concentrations of P than rice, wheat and corn, soybean requires more P than other crops to maintain its growth and development [31]. Previous studies have provided an understanding of the protein-coding genes and miRNAs involved in the response of soybean to phosphate starvation [14, 28, 29], but the role of lncRNAs in the response of soybean to LP stress has rarely been reported.

In this study, two contrasting genotypes of soybean, namely, Bogao (a LP-sensitive genotype) and Nannong 94156 (a LP-tolerant genotype), were used to investigate the regulatory mechanism of lncRNAs under P starvation. Using genome-wide high-throughput RNA sequencing (RNA-seq) technology, we identified and characterized a total of 4,166 lncRNAs that are responsive to LP stress in the roots of soybean seedlings, validated 14 lncRNAs by qPCR, and identified 525 differentially expressed (DE) lncRNAs related to the regulation of the tolerance of soybean to LP stress. We then performed GO and KEGG analyses and constructed an LP-responsive network to explore the putative functions of the identified lncRNAs. The results lay the foundation for obtaining a more in-depth understanding of the molecular mechanisms related to the roles of lncRNAs in response to LP stress. This study increases our knowledge of lncRNAs and provides new insights into the function of lncRNAs in LP stress.

## Results

### Identification and characterization of lncRNAs across two soybean genotypes under different P levels

To identify LP-responsive lncRNAs in soybean roots, we constructed 12 cDNA libraries from soybean root samples from two genotypes with contrasting responsiveness to LP stress, namely, Bogao (BG, a LP-sensitive genotype) and Nannong 94156 (NN94156, a LP-tolerant genotype), after exposure to high/normal phosphorus (HP, 500 µM, control) and low phosphorus (LP, 5 µM) conditions [32]. Three biological replicates of each condition were used to minimize the individual variation. The libraries were sequenced using the Illumina HiSeq 4000 platform, and 125-bp paired-end reads were generated. Approximately 1,087 million raw sequencing reads were generated from all 12 libraries, and each sample contained reads ranging from 75.5 to 100.7 million in number. After discarding adaptor sequences and low-quality reads (Q-value ≤ 20), more than 90 % of the total reads were retained [33]. We mapped these clean reads to the soybean reference genome sequence (*Wm82.a2.v1*). In total, 4,166 novel lncRNAs were predicted using the coding-noncoding index (CNCI) [34] and coding potential calculator (CPC) [35] under all tested conditions (Table S1).

**Fig. 1:**
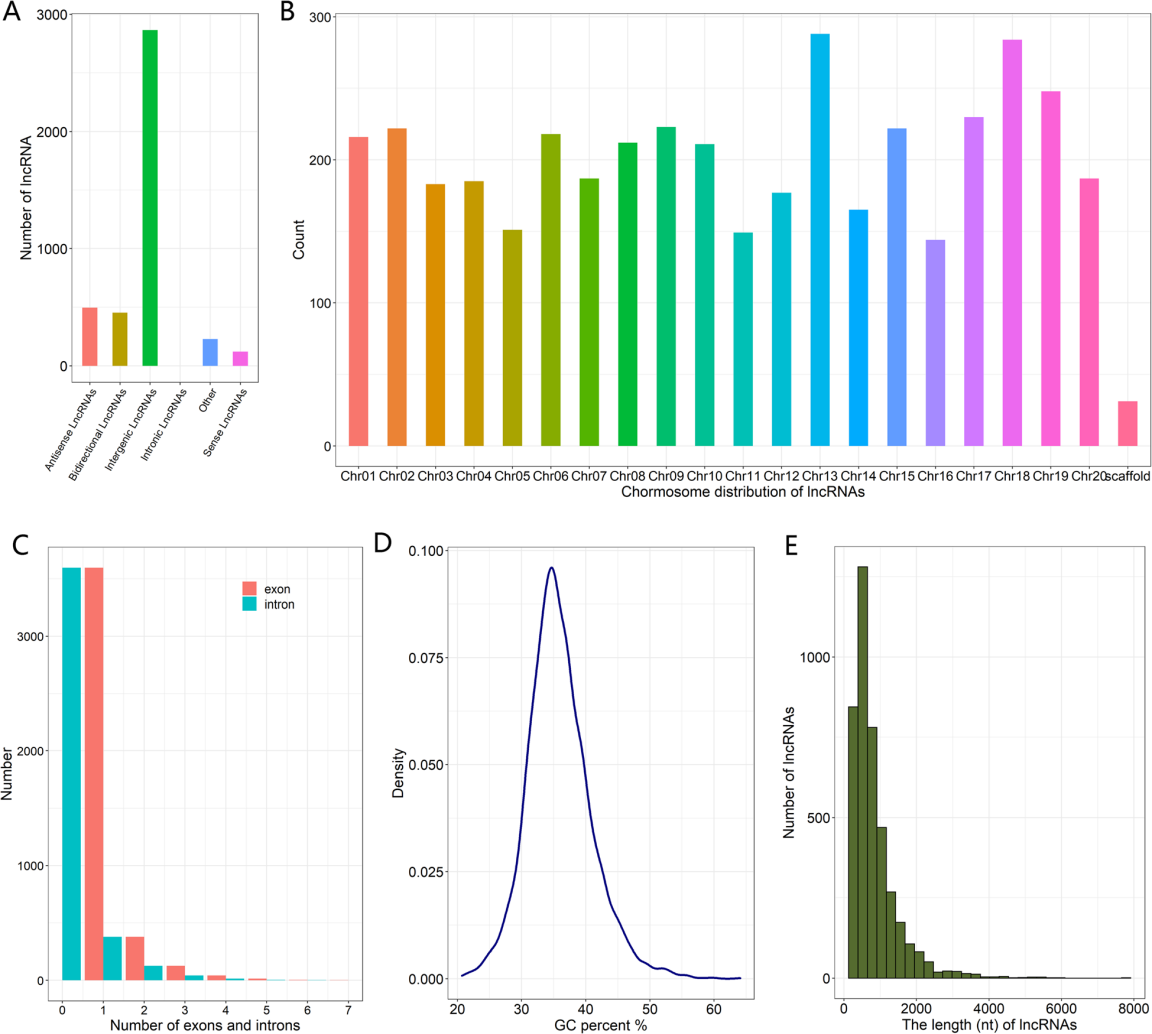
Identification and characterization of lncRNAs in soybean roots of two genotypes. **a** Number of identified lncRNAs in each type. **b** Chromosome-wise distribution of lncRNAs. **c** Numbers of predicted exons and introns in the lncRNAs. **d** GC percent (%) of the lncRNAs. **e** Sequence length distribution of lncRNAs

The classification of these lncRNAs showed that the majority (2,865, 68.77 %) of the 4,166 lncRNAs were located in intergenic regions, and the remaining 1,301 (31.23 %) resided within genic regions and included 454 bidirectional lncRNAs, 498 antisense lncRNAs, 121 sense lncRNAs, and 228 others that were not classified into these types (Fig. 1a). The type of lncRNA might be related to its functions; for example, overexpressed *LAIR* (a lncRNA transcribed from the antisense of the neighboring gene *LRK* cluster) regulates the expression of several *LRK* genes and increases the grain yield in rice [36]. We subsequently analyzed the chromosomal location of all the lncRNAs in the soybean genome. The distribution of the lncRNAs was uneven: chr13 and chr18 contained more than 250 lncRNAs, and chr05, chr11, and chr16 contained approximately 150 lncRNAs (Fig. 1b). In addition, we analyzed the number of exons and introns in each lncRNA transcript. Most of the lncRNAs contained one exon and no introns (3,597), and the number of exons and introns was as high as seven and six, respectively (Fig. 1c). The GC content of the lncRNAs varied greatly, with a range of 20.68–64.1 % and an average of 35.88 %.

The majority of lncRNAs have GC percent in the range of 30–45 % (Fig. 1d). A majority (94.43 %) of the lncRNAs were shorter than 2,000 nucleotides (Fig. 1e).

### Differentially expressed (DE) lncRNAs in two soybean genotypes under different P levels

**Fig. 2:**
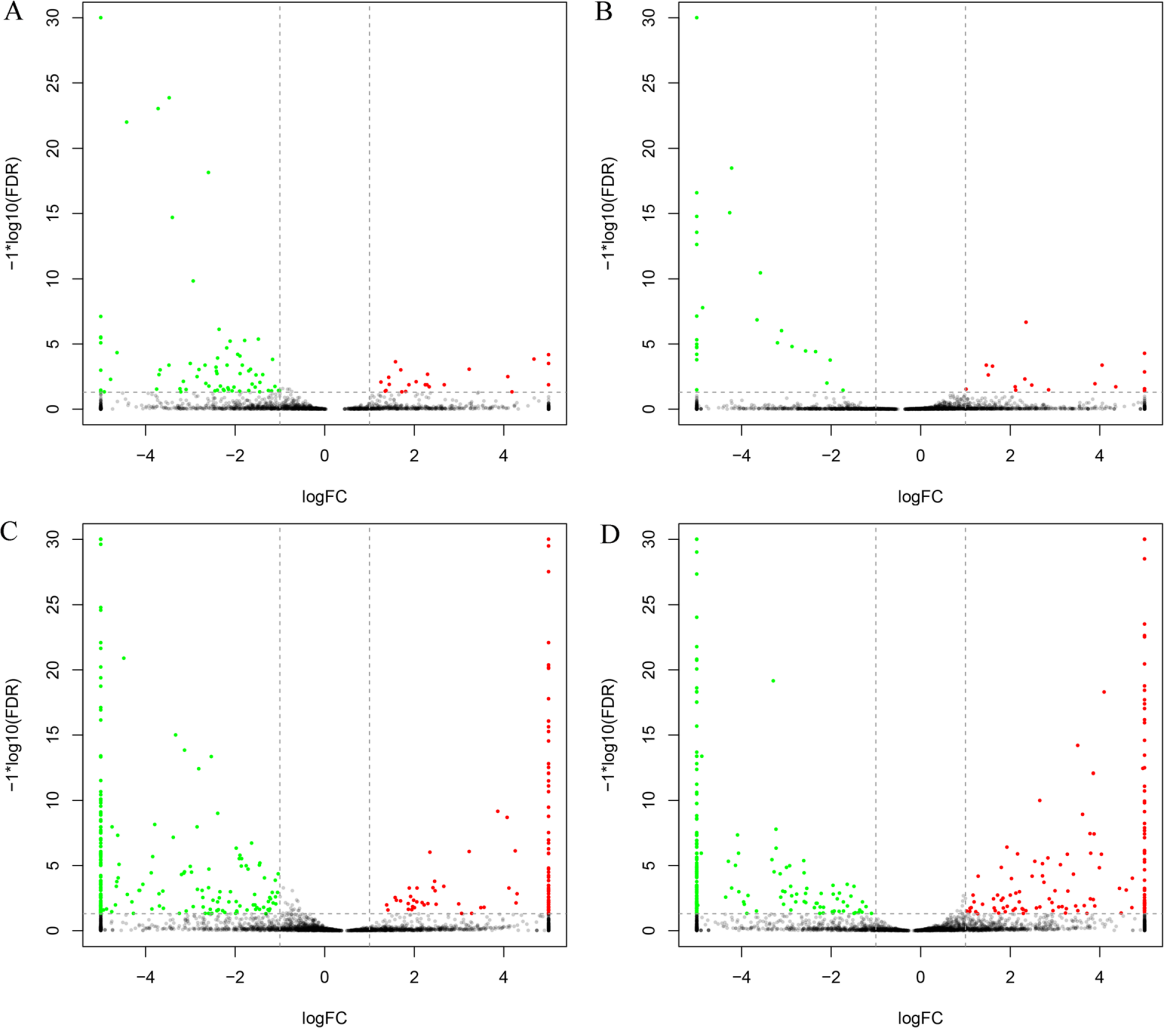
Volcano plots of differentially expressed (DE) lncRNAs in soybean roots under different P conditions. **a** HP_BR vs. LP_BR. **b** HP_NR vs. LP_NR. **c** HP_BR vs. HP_NR. **d** LP_BR vs. LP_NR. The red and green dots represent up- and downregulation, respectively. The x-axis represents the log2-fold change, and the y-axis represents the log_10_ *p*-value. *P*-value < 0.05 and |log_2_ fold change| > 1. HP and LP indicate high P and low P, respectively, and BR and NR represent roots of Bogao and NN94156, respectively

To identify the lncRNAs that are responsive to LP stress, we identified the differentially expressed (DE) transcripts of lncRNAs through pairwise comparisons between the two soybean genotypes under HP and LP conditions. The FPKM (fragments per kilobase of transcript per million mapped reads) values were used to evaluate the transcript abundance of lncRNAs. Differently expressed lncRNAs (referred to as DE lncRNAs hereafter) were defined as lncRNAs with Log_2_FC > 1 and FDR < 0.05. In total, 525 DE lncRNAs were identified among the two different genotypes under HP and LP conditions, and these included 116 DE lncRNAs between different P levels in the same genotype, 456 DE lncRNAs between different genotypes at the same P level, and 47 shared DE lncRNAs (Table S2). To identify the effect of LP stress on lncRNAs, we compared the DE lncRNAs of different genotypes under the same P condition and in the same genotype at different P levels (Fig. 2). As shown in the volcano plot, the LP treatment of Bogao and NN94156 resulted in more downregulated DE lncRNAs than upregulated DE lncRNAs, and the downregulated DE lncRNAs presented a more substantial change in differential expression than the upregulated DE lncRNAs (Fig. 2a and b). The number and fold change in expression of the upregulated and downregulated DE lncRNAs were relatively consistent in the Bogao and NN94156 genotypes under the same P level (Fig. 2c and d, Fig. S1).

**Fig. 3:**
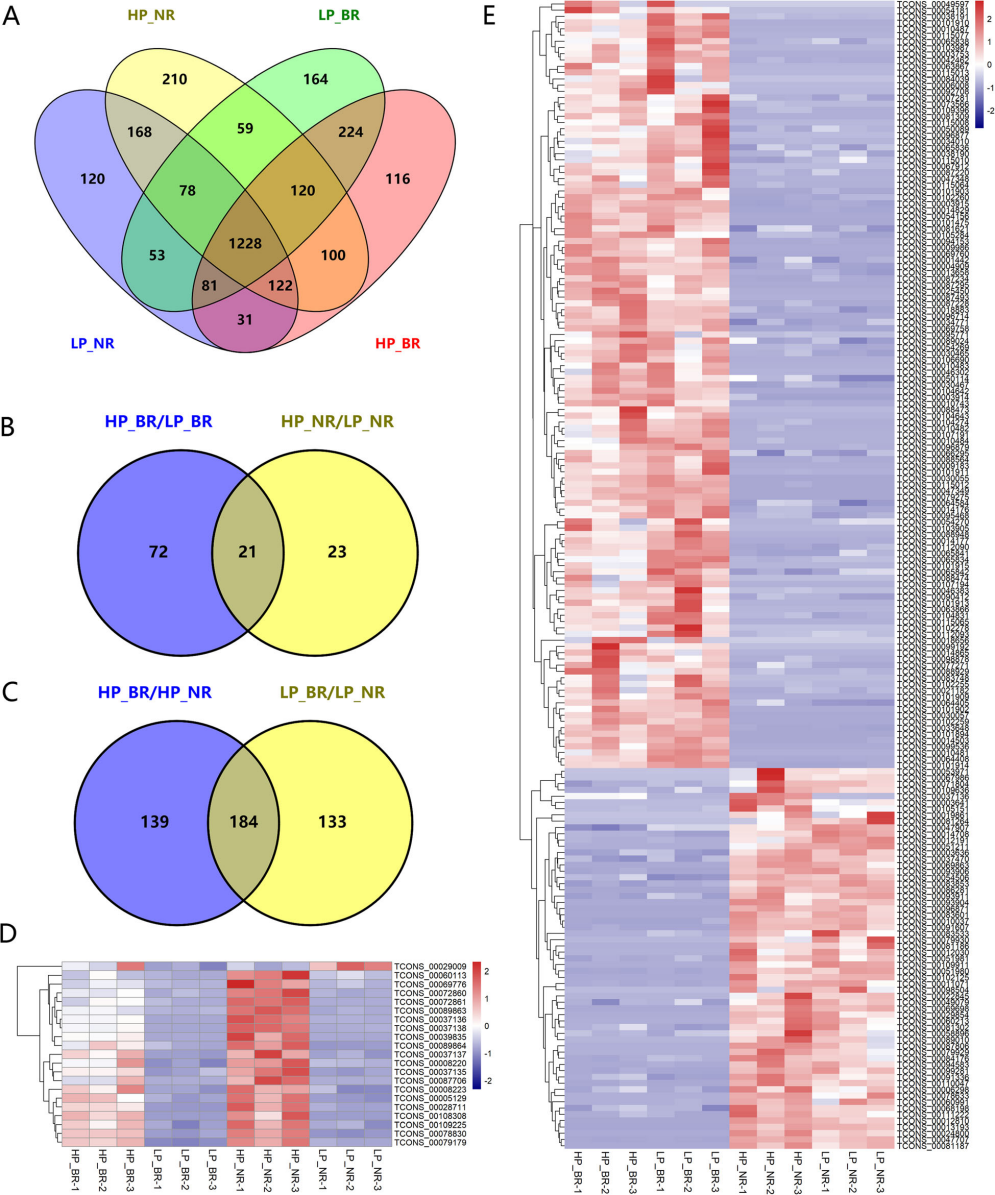
DE lncRNAs and expression patterns in soybean root plants under LP stress. **a** Venn diagram comparing the expressed lncRNAs in each root sample under different P levels. **b** Number of DE lncRNAs in the same genotype between different P levels. **c** Number of DE lncRNAs between different genotypes at the same P level. **d** Cluster analysis of the expression levels of common DE lncRNAs in the same genotype at different P levels. **e** Cluster analysis of the expression levels of common DE lncRNAs in different genotypes at the same P level

Because the two genotypes showed markedly different responses to LP stress, we performed a Venn diagram analysis to elucidate the DE lncRNAs between the two genotypes under LP conditions. The number of common and unique DE lncRNAs between the two genotypes is indicated in the Venn diagram (Fig. 3a). NN94156 and Bogao shared 21 common DE lncRNAs in the HP vs. LP comparisons, and Bogao exhibited more genotype-specific DE lncRNAs (72) than NN94156 (23) (Fig. 3b), which is consistent with the results shown in the volcano plot (Fig. 2a and b). We found that the 21 common DE lncRNAs in Bogao were all downregulated under LP conditions, whereas most of these downregulated lncRNAs (20, all except TCONS_00029009) were also downregulated in NN94156 (Fig. 3d). To determine whether the effect of LP stress on lncRNAs is related to genotype, we compared the changes in DE lncRNAs between Bogao and NN94156 under LP or HP conditions. The results identified 133 and 139 unique DE lncRNAs under the LP and HP conditions, respectively (Fig. 3c). The 184 common DE lncRNAs showed the same up- or downregulation trend: 123 were downregulated, and 61 were upregulated (Fig. 3e).

### Validation and quantification of lncRNAs

**Fig. 4:**
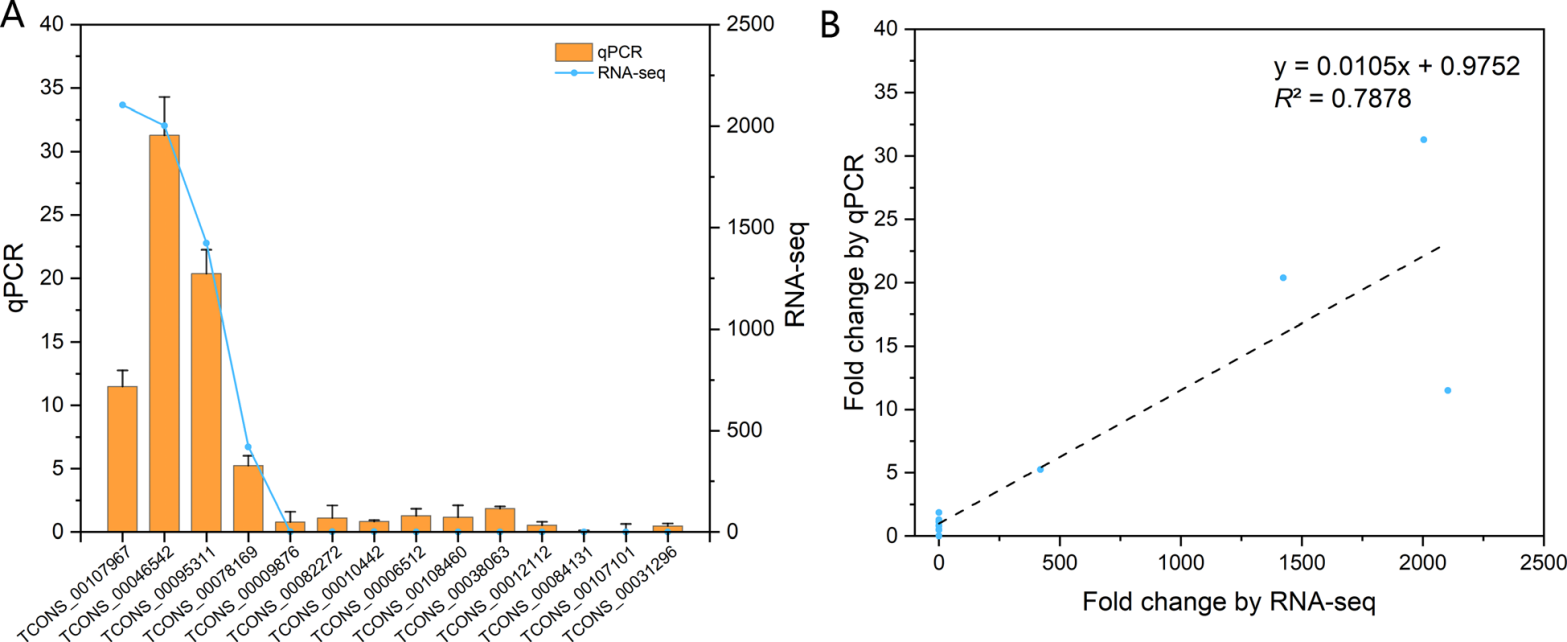
Confirmation of the expression patterns of lncRNAs by qPCR. **a** Fold change obtained by lncRNA-seq and qPCR (LP/HP). **b** Linear regression analysis of lncRNA-seq and qPCR data

To validate the expression of these LP-responsive lncRNAs, 14 lncRNAs were randomly selected and analyzed by quantitative PCR (qPCR). As shown in Fig. 4a, the expression patterns of the LP/HP lncRNAs determined by RNA-seq and qPCR were relatively consistent and presented similar trends. Both the qPCR and RNA-seq assays revealed a positive correlation in the expression fold-change with an *R*^2^ of 0.7878 (Fig. 4b), which indicated the robustness of our analysis and the reliability of the lncRNA expression patterns identified in the current study. These findings confirm that these lncRNAs are responsive to LP stress in soybean roots.

### Functions and expression patterns of DE lncRNAs and their target genes

To reveal the potential functions of the differentially expressed lncRNAs under LP stress in two contrasting genotypes, we predicted the candidate targets of *cis*-, *trans*- and antisense-acting DE lncRNAs. In total, 785 targets of 374 DE lncRNAs were identified, and for 960 pairs, one lncRNA might have several targets and/or one mRNA target might be targets of several lncRNAs (Table S3). To explore the putative functions of DE lncRNAs, we analyzed the Gene Ontology (GO) terms (Table S4) and Kyoto Encyclopedia of Genes and Genomes (KEGG) pathways of the putative target genes (Table S5).

**Fig. 5:**
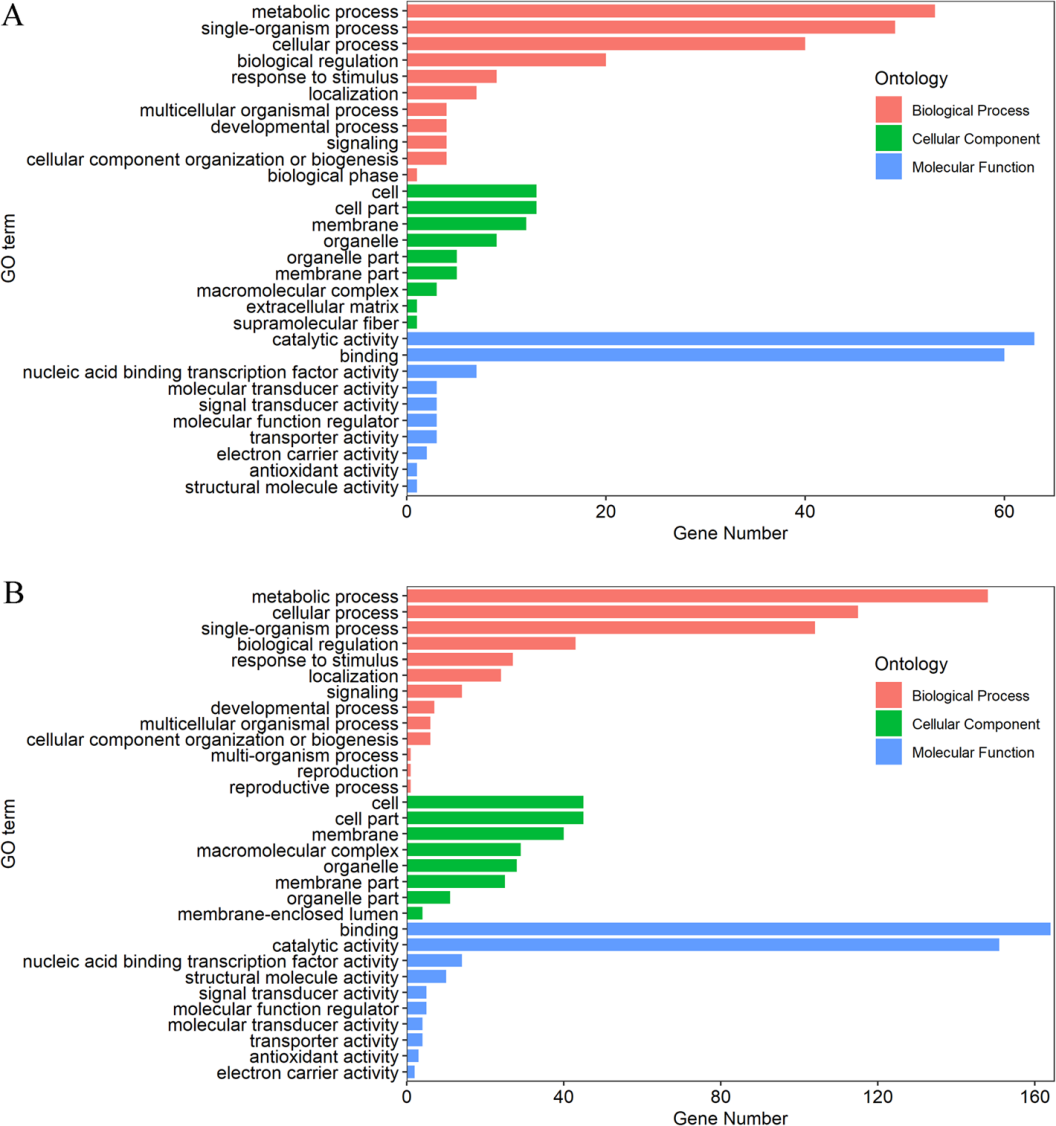
Gene ontology (GO) enrichment of DE lncRNAs targets. **a** Targets of DE lncRNAs in the same genotype between different P levels. **b** Targets of DE lncRNAs between different genotypes at the same P level

The GO analysis of DE lncRNAs in one genotype at different P levels revealed that 403 GO terms (195 in the biological process category, 146 in the molecular function category, and 62 in the cellular component category) were significantly enriched (*P < 0.05*) (Fig. 5a). The analysis of the DE lncRNAs in Bogao or NN94156 exposed to the same P level showed that 1,086 GO terms (497 in the biological process category, 362 in the molecular function category, and 227 in the cellular component category) were significantly enriched (*P < 0.05*) (Fig. 5b). Although the numbers of GO terms in the two genotypes were different, their trends were relatively similar. In brief, the most significant GO terms related to biological process were metabolic process, single-organism process, and cellular process, and the analysis of molecular functions revealed that catalytic activity and binding were the important significantly enriched GO terms. In addition, cell, cell part, membrane and organelle were the most important significant terms belonging to the cellular component categories. Taken together, these results show that these lncRNAs might play roles in a variety of biological processes that are responsive to LP stress.

**Fig. 6:**
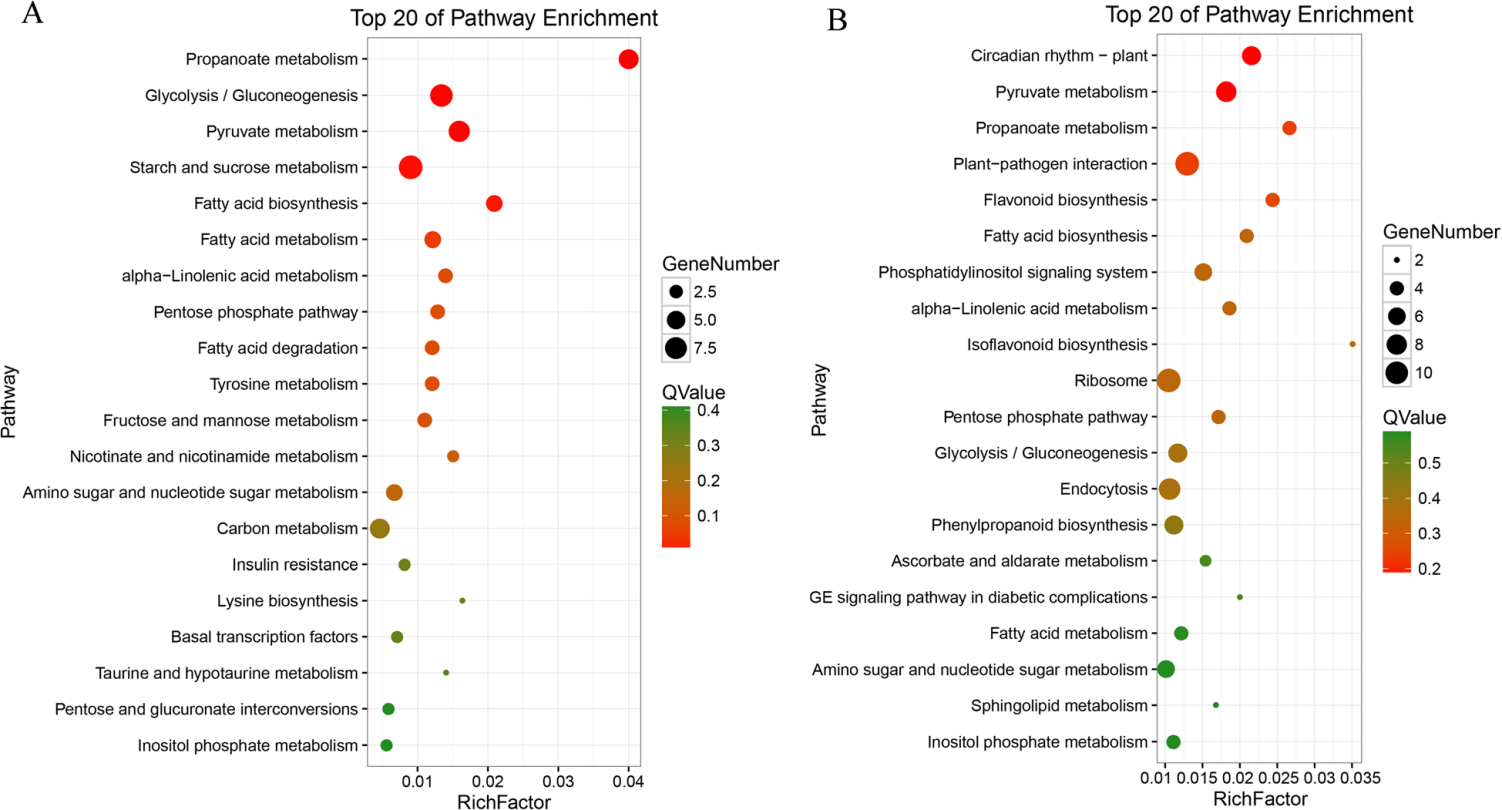
KEGG enrichment of DE lncRNA targets. **a** Targets of DE lncRNAs in the same genotype between different P levels. **b** Targets of DE lncRNAs between different genotypes at the same P level

We subsequently analyzed the enrichment of the predicted target genes of DE lncRNAs in KEGG pathways (Table S5). The targets of DE lncRNAs in the same genotype between different P levels were enriched in 42 KEGG pathways, including several KEGG pathways related to carbohydrate metabolism, lipid metabolism, and amino acid metabolism (Fig. 6a). For example, propanoate metabolism, glycolysis/gluconeogenesis, pyruvate metabolism, starch and sucrose metabolism, and the pentose phosphate pathway belong to carbohydrate metabolism, and fatty acid biosynthesis, alpha-linolenic acid metabolism and fatty acid degradation are lipid metabolism pathways. The analysis of targets of DE lncRNAs between Bogao and NN94156 under the same conditions (HP and LP) showed that 74 KEGG terms were enriched, and these included environmental adaptation, carbohydrate metabolism, biosynthesis of other secondary metabolites, lipid metabolism, and signal transduction. Among the top 20 enriched pathways (Fig. 6b), circadian rhythm-plant and plant-pathogen interactions belong to environmental adaptation and were significantly enriched (Q-values < 0.05). Terms related to three secondary metabolite pathways, including flavonoid biosynthesis, isoflavonoid biosynthesis, and phenylpropanoid biosynthesis, were enriched. These findings suggest that DE lncRNAs might regulate genes involved in many biological processes, including molecular metabolism, energy synthesis and signal transduction, in response to LP stress.

### Putative P-related lncRNAs based on miRNAs

miRNAs are endogenous noncoding RNAs with a size of 20 to 24 nucleotides that are generated from a single-stranded RNA precursor with a hairpin secondary structure. LncRNAs can be spliced by miRNAs into multiple small RNAs, and as a result, the function of lncRNAs can be regulated by miRNAs via posttranscriptional regulation. For example, *miR399* is the first miRNA that was found to be upregulated specifically by P deficiency and rapidly decreased after P readdition, and certain miRNA families are responsive to P deficiency in various species [37].

**Fig. 7:**
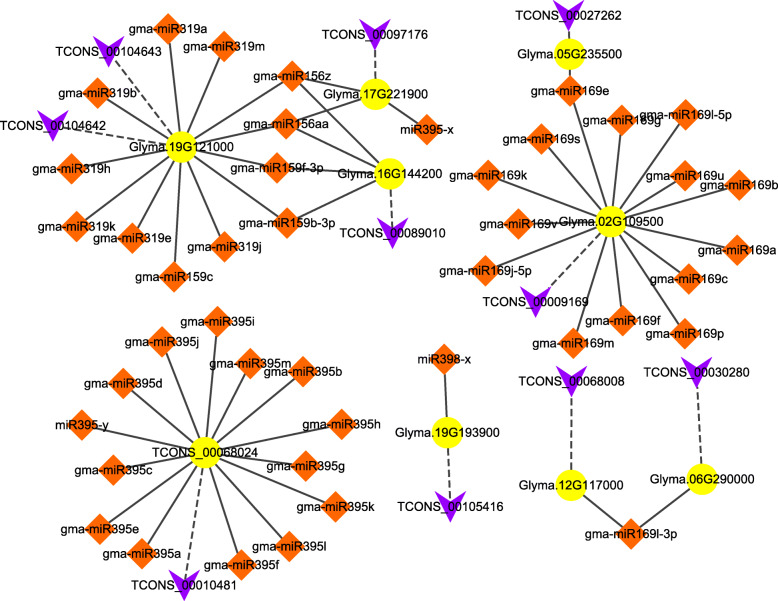
Prediction of lncRNA-mRNA-miRNA networks associated with LP stress. The mRNA-lncRNA and miRNA-mRNA interaction networks were constructed based on P-related miRNAs. The yellow circles denote mRNAs, the orange rhombuses denote miRNAs, and the purple V shapes indicate lncRNAs. The solid line represents a miRNA-mRNA interaction, and the dashed line indicates a lncRNA-mRNA interaction

Because our research mainly focused on LP stress, various targets, including lncRNAs and mRNAs of P-related miRNAs, such as *miR399*, *miR827*, *miR395*, *miR319*, *miR156*, *miR159*, *miR166*, *miR169*, *miR398* and *miR447* [27, 37, 38], were selected. The lncRNA *TCONS_00090111* was identified as a target of five miRNAs, namely, *gma-miR156aa*, *gma-miR156z*, *gma-miR159b-3p*, *gma-miR159c*, *and gma-miR159f-3p*. Similarly, *TCONS_00015352* was predicted as a target of *miR447-y* (Table 1). The P-related miRNA targets were then predicted, and we found that nine mRNAs were also targets of lncRNAs (Table S6). As shown in Fig. 7, *Glyma.19G121000*, *Glyma.02G109500* and *TCONS_00068024* (a novel identified mRNA) were targets of lncRNAs and several miRNAs. *Glyma.06G290000* and *Glyma.12G117000* were both annotated as ethylene-responsive transcription factor 9-like mRNAs and were targets of the lncRNAs *TCONS_00030280* and *TCONS_00068008*, respectively. Both genes were also targets of *gma-miR169l-3p*, which is a P-related miRNA [39]. Another example is *Glyma.19G193900*, which was predicted to be purple acid phosphatase 22-like, is the target of *TCONS_00105416* and *miR398-x*, which belong to *miR398* and have a demonstrated role in coping with P starvation stress [37].

**Table 1:** LncRNAs identified as targets of P-related miRNAs

miRNA	Target lncRNA
gma-miR156aa	TCONS_00090111
gma-miR156z	TCONS_00090111
gma-miR159b-3p	TCONS_00090111
gma-miR159c	TCONS_00090111
gma-miR159f-3p	TCONS_00090111

### Construction of the network of transcription factors (TFs) and P-related and plant hormone-associated lncRNAs and mRNAs

**Fig. 8:**
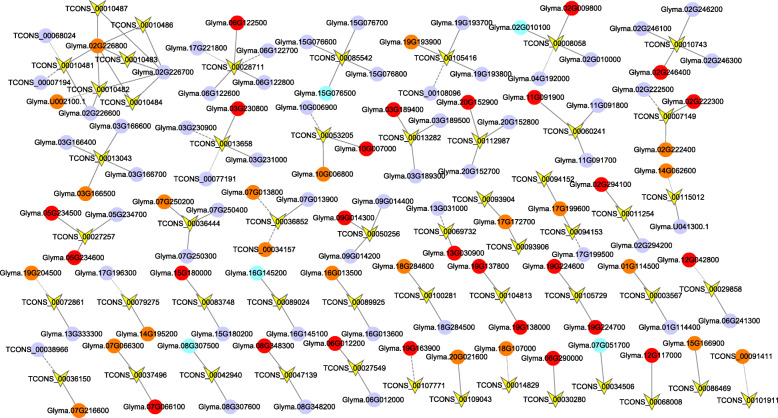
Prediction of lncRNA-mRNA networks of targets of interest, including TFs and P-related and plant hormone-associated genes. The yellow V shapes indicate lncRNAs, the circles denote mRNAs, the orange rhombuses denote miRNAs, and the purple V shapes indicate lncRNAs. The circles represent mRNA targets of lncRNAs, the red, orange, blue and purple colors indicate TFs, P-related genes, plant hormones and other genes targeted by lncRNAs. The solid and dots lines represent *cis-* and *trans-*acting lncRNA-mRNA interactions, respectively, and the dashed line indicates an antisense interaction. Eight ethylene-responsive transcription factors (*Glyma.19G138000*, *Glyma.10G007000*, *Glyma.19G163900*, *Glyma.02G294100*, *Glyma.06G290000*, *Glyma.12G117000*, *Glyma.15G180000*, and *Glyma.08G348300*) are indicated as TFs

TFs regulate a diverse group of genes during stress responses and are important components of gene regulatory networks, and many TFs belonging to some families have been proven to play an important role in the maintenance of P homeostasis, such as the phosphate starvation response (PHR), bHLH, WRKY, ZAT, and MYB [40]. The P-mediated regulation of the root system architecture is driven by the local perception of PO_4_^−^ at the root tip and involves changes in multiple plant hormones, such as auxin, gibberellins and ethylene, as well as hormonal changes coordinated with the root developmental responses to P availability [38]. P-related genes such as *PHO2* and *PHR1* play important roles in the P starvation response. To further study the function of lncRNAs in the responses of soybean roots to LP stress, we constructed a lncRNA-mRNA network of mRNAs of interest (including transcription factors and P-related and plant hormone targets) and corresponding lncRNAs according to the GO, KEGG and functional annotations of the target genes (Table S7). As shown in Fig. 8, the lncRNA-mRNA network consisted of 52 lncRNAs and 109 targets in total. Twenty-three lncRNAs might be involved in the regulation of gene transcription because their target genes have transcription factor activity; in addition, three of the lncRNAs have two TF targets each, and 20 lncRNAs only have one TF target. The number of lncRNA targets varied from one to five, and *Glyma.02G226800* and *Glyma.02G226700* were targets of five and four lncRNAs, respectively. Twenty-six TFs belong to diverse families, such as MYB, bHLH, NAC, and AP2, and among these, MYB and bHLH reportedly play roles in the maintenance of P homeostasis [40]. Interestingly, we found that eight lncRNAs might be involved in ethylene regulation because their targets were annotated as ethylene-responsive TFs. Among the P-related genes, *Glyma.17G172700* and *Glyma.19G193900* were annotated as purple acid phosphatases (PAPs), and *Glyma.20G021600* was predicted to be a phosphate transporter that is known as a PHT and is involved in LP stress.

## Discussion

LncRNAs play important roles in a wide range of biological processes, particularly in plant reproductive development and responses to stresses [41]. However, little is known about their roles in LP stress, which is a major limiting factor in plant growth and the agricultural industry. Here, we undertook a genome-wide analysis of lncRNAs in two contrasting soybean genotypes subjected to phosphate starvation.

The number of lncRNAs varies greatly across plant species. For example, 48,345 lncRNAs have been identified in the maize transcriptome [15], and 1,212 novel lncRNAs have been found in *Arabidopsis* seedlings grown under P-sufficient and P-deficient conditions [14]. In this study, 4,166 lncRNAs were identified, and these lncRNAs show most of the common features of lncRNAs reported in other plants, such as short length, single exons, and low GC percent, which might be responsible for the common and ancient evolutionary origin of lncRNAs. In addition, we found that the sequence length of several (70) lncRNAs in soybean roots was longer than 3,000 bp. This finding is similar to the results from previous studies, which showed that 285 lncRNAs were longer than 3,000 bp and that the length of 28 lncRNAs was longer than 10,000 bp [42], and these results indicate the existence of a small number of long lncRNAs in plants. The type of lncRNAs was also highly variable in plants. We identified more intergenic lncRNAs (2,865, 68.77 %) than other types, including antisense lncRNAs (498, 11.95 %); in contrast, the number of antisense lncRNAs was greater than that of other types in *Arabidopsis* [14]. We questioned whether the number of exons was related to the length of the lncRNA gene, and our analysis revealed that the length of lncRNAs with a large number of exons was not significantly increased.

To identify the lncRNAs that were responsive to P stress, we identified the DE transcripts of the lncRNAs through pairwise comparisons between the two soybean genotypes under HP and LP conditions, and a total of 525 DE lncRNAs were identified among the two different genotypes under HP and LP conditions. As shown in Fig. 2a and b and Fig. S1, the number of DE lncRNAs under LP stress identified in Bogao was greater than that found in NN94156, which indicated that Bogao is more sensitive to LP treatment, and this result is consistent with our previous findings that Bogao and NN94156 are P-sensitive and P-tolerant genotypes, respectively [30, 33]. Furthermore, our previous results revealed that all mRNAs, circular RNAs and lncRNAs in Bogao are more sensitive to LP stress than those of NN94156 [30, 33]. The two genotypes showed substantially different responses to LP stress; NN94156 and Bogao shared 21 common DE lncRNAs in the HP vs. LP comparisons, and Bogao exhibited more genotype-specific DE lncRNAs (72) than NN94156 (23) (Fig. 3b). Most of the common DE lncRNAs were constitutively downregulated in the two genotypes, which indicated that the biological mechanisms of lncRNAs involved in basal responsiveness to LP stress were conserved in both soybean genotypes. Although a similar trend in their response to LP stress was found for these shared lncRNAs, the degree of the change in expression was significantly different (Fig. 3d and e). To determine whether the effect of LP stress on lncRNAs is related to genotype, we compared the changes in DE lncRNAs between Bogao and NN94156 under LP or HP conditions. We identified 317 DE lncRNAs (181 downregulated, 136 upregulated) under LP conditions, which suggested that these lncRNAs were constitutively but differentially expressed between the two genotypes under LP conditions. The 133 DE lncRNAs unique to LP conditions might play a role in LP tolerance. In contrast, 139 lncRNAs were differentially expressed in the two genotypes only under HP conditions, which suggested that their differential expression is specific to LP stress.

Studies have shown that lncRNAs can directly bind to mRNAs by affecting the translation, shearing, and degradation of mRNAs and can also indirectly influence the expression of mRNAs [17]. Thus far, the mechanism underlying the interaction between lncRNAs and mRNAs has not been clarified. To reveal the potential functions of the DE lncRNAs under LP stress in the two contrasting genotypes, we predicted the candidate targets of the DE lncRNAs and then analyzed the GO terms and KEGG pathways of their putative target genes (Table S5). The analysis of the DE lncRNAs in one genotype at different P levels and DE lncRNAs in Bogao or NN94156 exposed to the same P level revealed that 403 and 1,086 GO terms and 42 and 74 KEGG pathways were significantly enriched (*P < 0.05*), respectively (Fig. 5). Our enrichment results showed that LP stress is a complex regulatory network involved in diverse biological processes, such as lipid metabolic processes, catalytic activity, cell membrane formation, signal transduction, and nitrogen fixation (Figs. 5 and 6), and these findings are supported by previous studies focusing on LP in soybean [33, 43]. Previous research has shown that *NtMYB12* acts as a phosphorus starvation response enhancement factor and regulates *NtCHS* and *NtPT2* expression, which results in increased flavonol and P accumulation and enhances tolerance to LP stress [44]. In this study, targets of lncRNAs were enriched in various KEGG pathways, including flavonoid, isoflavonoid and phenylpropanoid biosynthesis, which indicates that these lncRNAs might be involved in the synthesis of secondary metabolites to regulate P-responsive genes, but this hypothesis needs further research.

LncRNAs can be spliced by miRNAs into multiple small RNAs, and as a result, the function of lncRNAs can be regulated by miRNAs via posttranscriptional regulation [37]. The targets of 10 P-related miRNAs (*miR399*, *miR827*, *miR395*, *miR319*, *miR156*, *miR159*, *miR166*, *miR169*, *miR398* and *miR447*) were predicted, and nine of them were also targets of lncRNAs (Table S6). Two lncRNAs, *TCONS_00030280* and *TCONS_00068008*, exhibited shared mRNA targets (*Glyma.06G290000* and *Glyma.12G117000*, annotated as ethylene-responsive transcription factor 9-like) with the P-related *miR169 l-3p* (Fig. 7). This finding was further supported by our recent study, which showed that NN94156 has the ability to tolerate LP stress via ethylene regulator-mediated enhanced P uptake and use efficiency in roots [32]. Therefore, lncRNAs might be partly involved in ethylene-mediated LP stress tolerance, and both genes are candidate genes that merit further investigation to gain further understanding of the involvement of lncRNAs in LP stress tolerance. *Glyma.19G193900*, which was predicted to be purple acid phosphatase 22-like, is the target of *TCONS_00105416* and *miR398-x*, which belong to *miR398* and have a demonstrated role in coping with P starvation stress [37]. PAPs are widely recognized as an adaptation strategy used by plants in response to P deficiency, and the secretion of PAPs plays important roles in P acquisition [45]. The identification of other genes using our preliminary scenario suggested that lncRNAs are involved in the response to LP stress through the manipulation of genes with a variety of functionalities, and many of these genes might also be cotargets of P-associated miRNAs. A detailed investigation of these genes might result in an increased understanding.

The enhancement of root hair production, which increases the root surface area for nutrient uptake, is a typical adaptive response of plants to phosphate starvation [46]. Ethylene plays an important role in root hair development induced by P starvation by controlling root hair elongation [38]. According to the GO, KEGG and functional annotations of target genes of DE lncRNAs, TFs and P-related and plant hormone targets were selected to construct a lncRNA-mRNA network (Fig. 8). Interestingly, we found that eight lncRNAs might be involved in ethylene regulation because their targets were annotated as ethylene-responsive TFs. Ethylene-responsive TFs belong to the APETALA2 (AP2)/ethylene response factor (ERF) family, which exists widely in plants. In *Arabidopsis*, the AP2/ERF TF superfamily comprises 147 members, and AP2/ERF proteins are known to regulate the responses of plants to various biotic and abiotic stresses and developmental processes. RNA interference and overexpression of *AtERF070* (AT1G71130), an ethylene response factor, results in alterations in the morphophysiological traits of roots and changes in the expression of a number of P starvation-responsive genes, which suggests a potential role for this TF in the maintenance of P homeostasis [40]. In addition to ethylene, auxin, GA and salicylic acid might be involved in the response to LP stress in soybean. The induction and secretion of acid phosphatases (APases) is considered an important strategy for improving plant growth under conditions of low inorganic phosphate. PAPs are an important class of plant APases that can be secreted into the rhizosphere to utilize organic phosphorus for plant growth and development [47]. Among the P-related genes, *Glyma.17G172700* and *Glyma.19G193900* were annotated as PAPs. *Glyma.20G021600* was predicted to be a phosphate transporter that is known as a PHT and has important roles in P acquisition, allocation, and signal transduction. We speculate that their corresponding lncRNAs might have similar functions and are involved in LP stress.

## Conclusions

The main aim of this research was to identify the potential lncRNA-related responses to LP stress in soybean and the differences in lncRNA responses between genotypes with different P efficiencies. Our results identified a total of 4,166 lncRNAs, including 525 DE lncRNAs, using roots of two representative genotypes under HP and LP conditions. LP stress can alter the genome-wide expression levels of lncRNAs, particularly in the P-sensitive genotype Bogao. These findings might provide a first look at the landscape of lncRNAs in soybean in response to LP stress. Moreover, we identified several promising lncRNAs that might have potential value for further analysis of the mechanism underlying the response of soybean to LP stress. Overall, this study enriches the knowledge concerning lncRNAs and provides some clues for exploring the function of lncRNAs in the response of soybean to LP stress.

## Methods

### Plant materials and growth conditions

The LP-tolerant genotype Nannong 94156 (NN94156) and the LP-sensitive genotype Bogao were provided by Soybean Research Institute, Nanjing Agricultural University, China, and grown hydroponically as described previously [43]. In brief, the seeds were surface-sterilized, germinated and grown in an artificial intelligence climate chamber at 28/20°C with a 10-h light/14-h dark photoperiod. When the two cotyledons had fully expanded, the soybean seedlings were transplanted into modified half-strength Hoagland’s nutrient solution (pH 5.8, 500 µM, KH_2_PO_4_, sufficient P, HP). Three days later, half of the seedlings were transferred to Hoagland’s nutrient solution with a low amount of P (5 µM P, LP), and the other half of the seeds were maintained under P-deficient conditions. The soybean plants were placed in the hydroponics box using a completely randomized block design. The solution was replenished every 3 d, and 10 d after the plants were transferred to the P-deficient conditions, three independent biological replicates of the roots of seedlings were collected (12 samples in total) and stored at -80 °C for total RNA extraction.

### RNA extraction, library construction, and Illumina sequencing

RNA extraction and purity, library construction, and Illumina sequencing were performed according to Lv et al. [33]. Briefly, after total RNA extraction, rRNAs were removed using an Epicenter Ribo-Zero rRNA Kit (Epicenter, USA, cat: MRZSR116), and 1 µg of rRNA-depleted RNA per sample was used to generate sequencing libraries according to the manual provided by Gene Denovo Biotechnology Co. (Guangzhou, China). The qualified libraries were then constructed and sequenced using an Illumina HiSeq 4000 platform. The 12 gene expression libraries were named HP-NR-1, HP-NR-2, HP-NR-3; LP-NR-1, LP-NR-2, LP-NR-3; HP-BR-1, HP-BR-2, HP-BR-3; LP-BR-1, LP-BR-2, and LP-BR-3. The lncRNAs, mRNAs, miRNAs and circRNAs were sequenced simultaneously using the same samples and corresponding published results [32, 33, 48].

### Identification of lncRNAs

The raw data were preprocessed to filter out adapters, reads containing more than 10 % unknown nucleotides and reads containing more than 50 % low-quality (Q-value ≤ 20). The obtained clean reads were then aligned to the soybean reference genome Williams 82 *Wm82.a2.v1* using the splice read aligner TopHat2 [49]. Transcripts longer than 200 bp with exon numbers greater than 1 were selected. Two software programs, CNCI (coding-noncoding index) [34] and CPC (coding potential calculator) [35], were used to assess the protein-coding potential of the transcripts using the default parameters. The intersection of both nonprotein-coding potential results was selected as long noncoding RNAs (lncRNAs). The lncRNA differential expression analysis between two different groups was performed using DESeq [50] software. Transcripts with a false discovery rate (FDR) below 0.05 were considered DE genes. Venn diagrams were generated using Venny 2.1.0 (https://bioinfogp.cnb.csic.es/tools/venny/index.html).

### Quantitative PCR (qPCR) validation of lncRNAs

To validate the expression data obtained by RNA-seq, 14 lncRNAs were selected randomly for quantitative PCR (qPCR) analysis. qPCR experiments were performed with an ABI 7500 system (Applied Biosystems, Foster City, CA, USA). Each PCR contained 10 µL of qPCR SYBR MIX (Toyobo, USA), 50 ng of cDNA and 0.5 µL of 10 µmol L^− 1^ gene-specific primers. The PCR amplification procedure was 95 °C for 5 min followed by 40 cycles of 95 °C for 15 s and 60 °C for 60 s. The *tubulin* (GenBank accession: AY907703) gene in soybean was used as an internal control, and samples in which the cDNA template was replaced by ddH_2_O were used as a negative control. This experiment was performed with three technical replicates and three biological replicates, and the relative expression of lncRNAs was analyzed using the 2^−ΔΔCT^ method [51]. The genes and their primers are listed in Table S8.

### Target gene prediction and functional analysis

We searched for coding genes 10 kb upstream and downstream of the identified lncRNAs and then predicted them as *cis*-acting lncRNAs targeting neighboring genes. Some antisense lncRNAs might regulate gene silencing, transcription and mRNA stability. RNAplex software [52] (https://www.tbi.univie.ac.at/RNA/RNAplex.1.html) was used to predict the complementary correlation of antisense lncRNAs and mRNAs, and mRNAs were predicted as antisense genes of lncRNAs. Another function of lncRNAs is the transregulation of coexpressed genes not adjacent to lncRNAs. The expression-related correlation between lncRNAs and protein-coding genes was analyzed to identify *trans*-acting genes of lncRNAs. These lncRNA target genes were functionally annotated using the GO (http://geneontology.org/) and KEGG (http://www.genome.jp/kegg/) databases.

### Analyses of lncRNAs and/or mRNAs with miRNAs

To find potential miRNA precursors, lncRNAs were aligned to miRBase, and those with more than 90 % similarity were selected. In addition, miRPare software, which is based on the SVM method, was also used for the prediction of miRNA precursors. The lncRNA-mRNA-miRNA network analysis was conducted as follows. First, 10 P-related miRNAs (*miR399*, *miR827*, *miR395*, *miR319*, *miR156*, *miR159*, *miR166*, *miR169*, *miR398* and *miR447*) were selected, and PatMatch software (v1.2) was used to predict the target genes of these miRNAs. Second, nine common target genes were found to exist among the lncRNA and miRNA targets. Third, the common target genes of lncRNAs and miRNAs were integrated to form a lncRNA-mRNA-miRNA network. The lncRNA-mRNA network analysis proceeded as follows. First, TFs and P-related and plant hormone-related mRNAs were selected as mRNAs of interest according to their GO, KEGG and functional annotations. Second, the lncRNAs corresponding to these mRNAs of interest were searched. Third, all targets of these lncRNAs were selected, including mRNAs of interest (TFs and P-related and plant hormone-associated genes), and non-interested targets were classified as none. Finally, a lncRNA-mRNA-miRNA network was constructed using Cytoscape 3.8.0 [53] software.

## Supplementary Information

**Additional file 1: Figure S1.** Number of up- and downregulated DE lncRNAs under LP and HP conditions in the two soybean genotypes.

**Additional file 2: Table S1.** Detailed information of identified lncRNAs in soybean roots.

**Additional file 3: Table S2.** DE lncRNAs in different genotypes and P levels.

**Additional file 4: Table S3.** The target mRNAs of DE lncRNA in two comparisons.

**Additional file 5: Table S4.** GO enrichment analysis of the targeted mRNAs of significantly DE lncRNAs in two comparisons.

**Additional file 6: Table S5.** KEGG pathway annotation of the predicted target mRNAs of DE lncRNAs in two comparisons.

**Additional file 7: Table S6.** The mRNAs as targets of miRNA and lncRNA.

**Additional file 8: Table S7.** The interested lncRNA-mRNA prediction for network construction.

**Additional file 9: Table S8.** List of primers used for qPCR of lncRNAs.

## Data Availability

The datasets generated and/or analyzed during the current study are available in the NCBI Sequence Read Archive (SRA) Database, accession number SRP233239 (https://www.ncbi.nlm.nih.gov/sra/?term=SRP233239).

## Abbreviations

lncRNAs: Long non-coding RNAs
ORF: Open reading frame
APA: Acid phosphatase activity
FDR: False Discovery Rate
DE: Differentially expressed
RNA-seq: RNA sequencing
FPKM: Fragments Per Kilobase of transcript per Million mapped reads
RSA: Root system architecture
P: Phosphorus
LP: Low-phosphorus
CNCI: Coding-non-coding index
CPC: Coding potential calculator
qPCR: Quantitative PCR
KEGG: Kyoto Encyclopedia of Genes and Genomes
GO: Gene Ontology
PAPs: Purple acid phosphatases
AP2/ERF: Apetala2/Ethylene Response Factor
TFs: Transcription factors
