# Supplementary figures and images for: Genome-wide analysis of long non-coding RNAs (lncRNAs) in two contrasting soybean genotypes subjected to phosphate starvation
Source: BMC Genomics. 2021 Jun 9;22:433. doi: 10.1186/s12864-021-07750-8 (PMC8191232; doi:10.1186/s12864-021-07750-8)

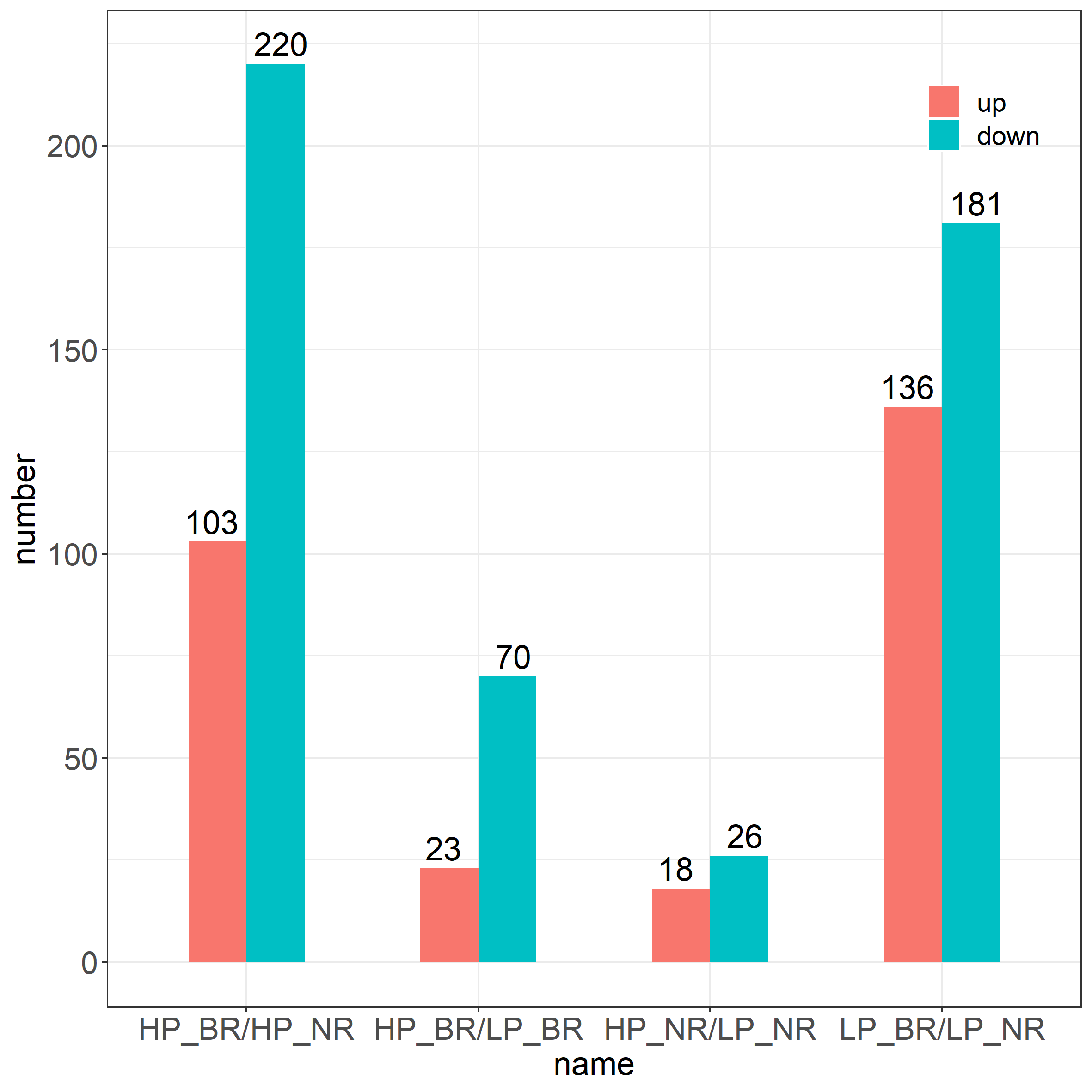

Supplement: Supplementary file 1 — Additional file 1: Figure S1. Number of up- and downregulated DE lncRNAs under LP and HP conditions in the two soybean genotypes. [file 12864_2021_7750_MOESM1_ESM.png]
